# Electrical conductivity of silver nanoparticle doped carbon nanofibres measured by CS-AFM†

**DOI:** 10.1039/c8ra04594a

**Published:** 2019-02-05

**Authors:** Wael Ali, Valbone Shabani, Matthias Linke, Sezin Sayin, Beate Gebert, Sedakat Altinpinar, Marcus Hildebrandt, Jochen S. Gutmann, Thomas Mayer-Gall

**Affiliations:** Deutsches Textilforschungszentrum Nord-West gGmbH Adlerstr. 1 D-47798 Krefeld Germany mayer-gall@dtnw.de jochen.gutmann@uni-due.de; Department of Physical Chemistry and Center for Nanointegration Duisburg-Essen (CENIDE), University Duisburg-Essen Universitätsstrasse 2 D-45141 Essen Germany

## Abstract

In this work, a pioneering study on the electrical properties of composite carbon nanofibres (CNFs) using current-sensitive atomic force microscopy (CS-AFM) has been demonstrated. CNFs are highly interesting materials which are usable in a wide array of applications *e.g.* electrode materials for biosensors, lithium ion batteries, fuel cells and supercapacitors. CNFs offer a high specific surface area and thus have a high contact area for charge transfer. CNFs can be produced using spinnable polyacrylonitrile (PAN) as a precursor for carbonisation. For the purpose of developing efficient CNFs with high conductivity and power density, silver nanoparticle (AgNPs)-containing PAN solutions were electrospun to form composite nanofibres which was followed by heat treatment. The applied voltage of the spinning setup and the content of both PAN and the silver nanoparticles in the spinning solution were varied in order to study their influence on the morphology and the electrical properties of the nanofibres. The resultant morphologies and fibre diameters were determined by scanning electron microscopy (SEM). The formation of silver nanoparticles was characterised in solution by UV-visible absorption spectroscopy and dynamic light scattering (DLS), while energy-dispersive X-ray spectroscopy (EDX) and transmission electron microscopy (TEM) were carried out to investigate the presence as well as the average diameter of the AgNPs. The electrical properties of the CNFs were investigated using CS-AFM. This technique gives us the possibility to explore the electrical properties of single fibers and hence derive relationships between the structural features and the electrical properties. Our results show that the composite CNFs have a higher electrical conductivity than the neat CNFs and both the average diameter of the fibers and the electrical conductivity increase with an increasing AgNP content.

## Abstract 2

In this work, a pioneering study on the electrical properties of composite carbon nanofibres (CNFs) using current-sensitive atomic force microscopy (CS-AFM) has been demonstrated.

## Graphical abstract

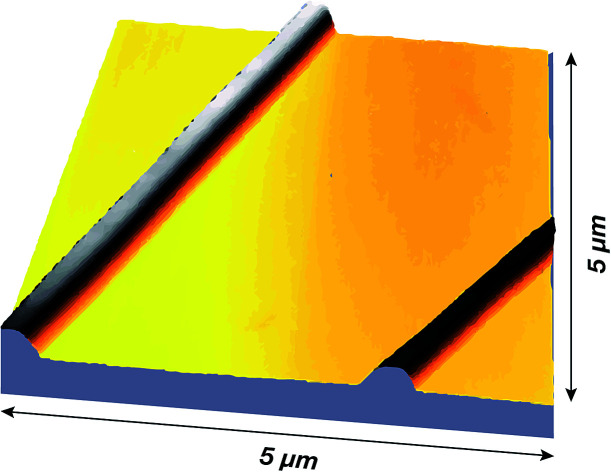

## Introduction

**Scheme 1:**
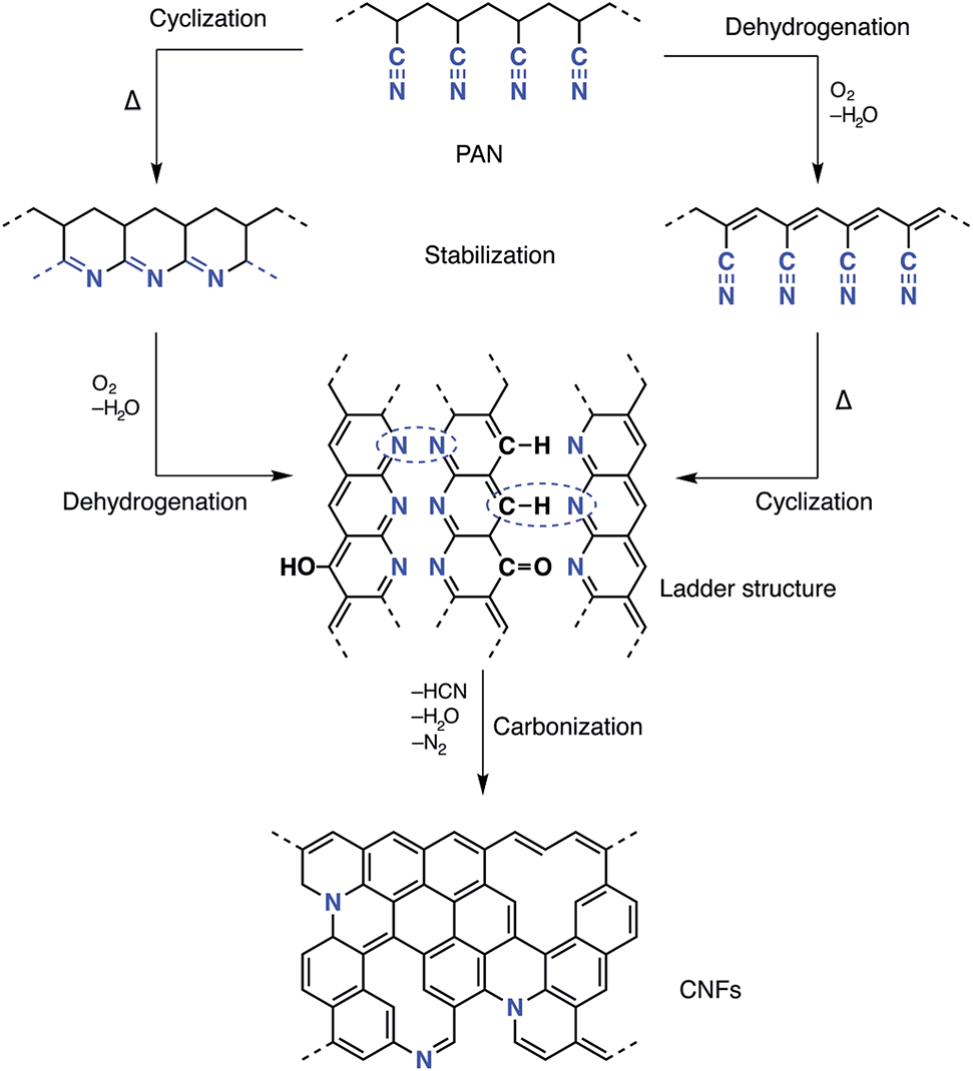
Schematic of the proposed chemistry of PAN stabilisation and carbonisation.

Recent progress in nanoscience and nanotechnology has paved the way for the design of polymer-based nanofibre materials that have superior properties in comparison to normal fibres. Particularly, carbon nanofibres (CNFs) have received much attention due to their broad potential applications in many fields, including sensors and electrical devices, energy conversion and storage, and catalysts or catalyst support materials.^1–10^ Among several precursors fulfilling the requirements for producing CNFs, polyacrylonitrile (PAN) is most commonly used, not only because of its high carbon yield compared to other polymers (>50%), but also because of the flexible and stable structure of the final products as well as its low price.^11–13^ Several methods have been reported for the fabrication of precursor carbon fibres including laser ablation, arc-discharge, vapour growth, chemical vapour deposition, pressurised gyration and electrospinning.^13–15^ Electrospinning is a well-established technique that has emerged with great potential to generate a wide variety of polymeric fibres in numerous research areas. Electrospinning has the ability to fabricate continuous fibres with diameters ranging from submicron to nanometer size using a huge range of polymeric materials, as well as composites, semiconductors and even ceramic materials.^12,16,17^ A typical electrospinning set-up, at laboratory level, consists of a high-voltage power supply (up to 30 kV), a syringe with a flat tipped metallic needle (spinneret) and a conducting collector. More than 90% of commercial carbon fibres are currently produced by heat treatment processes of PAN precursors under controlled conditions which affect oxidative stabilisation and subsequent carbonisation.^18^ The effect of thermal conditions on both the conversion of the PAN precursor and the formation of the carbon fibres has been investigated intensively in the last few decades. The oxidative stabilisation process is the most important and complicated step, since different gas–solid phase cascade chemical reactions take place and the molecular structure of the carbon fibre is ordinated in this stage.^19–21^ Formation of a conjugated ladder structure follows the cyclisation of the nitrile groups and the dehydrogenation or aromatisation reactions as shown in Scheme 1. The subsequent carbonisation process includes crosslinking of the cyclised polymer structure through denitrogenation under an inert atmosphere resulting in a graphitic structure.

Owing to the high surface area-to-volume ratio of the electrospun fibres and their unique tailor-made properties, PAN-based CNFs are useable for different electronic devices. However, owing to the large electrical resistivity resulting from the low electrochemical activity and due to the limitation of the surface area, the power density of the PAN-based CNFs is poor.^22,23^ Many efforts have been made to improve the electrical conductivity of PAN-based CNFs. Kim and Yang reported the chemical activation of CNFs electrospun from PAN using ZnCl_2_ with excellent performance as supercapacitors.^22^ Wang *et al.* demonstrated that the electrical conductivity increased with increasing carbonisation temperature as the graphite domain size increased.^24^ Ra *et al.* developed a porous carbon nanofibre paper by one-step carbonisation/activation of a PAN-based nanofibre paper under a CO_2_ atmosphere.^9^ The paper was used as a supercapacitor electrode without any binder or percolator. One effective approach to enhance the electrochemical properties and enlarge the reactive surface area of CNFs is to incorporate metal nanoparticles into polyacrylonitrile fibres.^25–28^ This approach has attracted much attention due to the large interfacial area as well as the synergistic effect of the resulting nanocomposites. In this context, it is well known that silver nanoparticles (AgNPs) possess interesting catalytic and electrochemical properties, and thus materials containing silver are promising candidates in many scienctific activities. Many agents have been used to form silver nanoparticles *via* the reduction of silver ions (Ag^+^).^29^ Pastoriza-Santos was the first to show that *N*,*N*-dimethylformamide (DMF) is a powerful reducing agent for Ag^+^.^30^ The advantage of this method is that DMF can play a dual role as a solvent for the PAN precursor as well as an effective agent to reduce silver ions (Ag^+^) resulting in silver nanoparticles (AgNPs), and this solution can be directly electrospun into PAN nanofibres.^31^ The mechanism of DMF oxidation proposed by Liz-Marzán involves the formation of carbamic acid, which can be decomposed as shown in the following equations:^30^HCON(CH_3_)_2_ + 2Ag^+^ + H_2_O → 2Ag^+^ + (CH_3_)_2_NCOOH + 2H^+^(CH_3_)_2_NCOOH → CO_2_ + (CH_3_)_2_NH

**Fig. 1:**
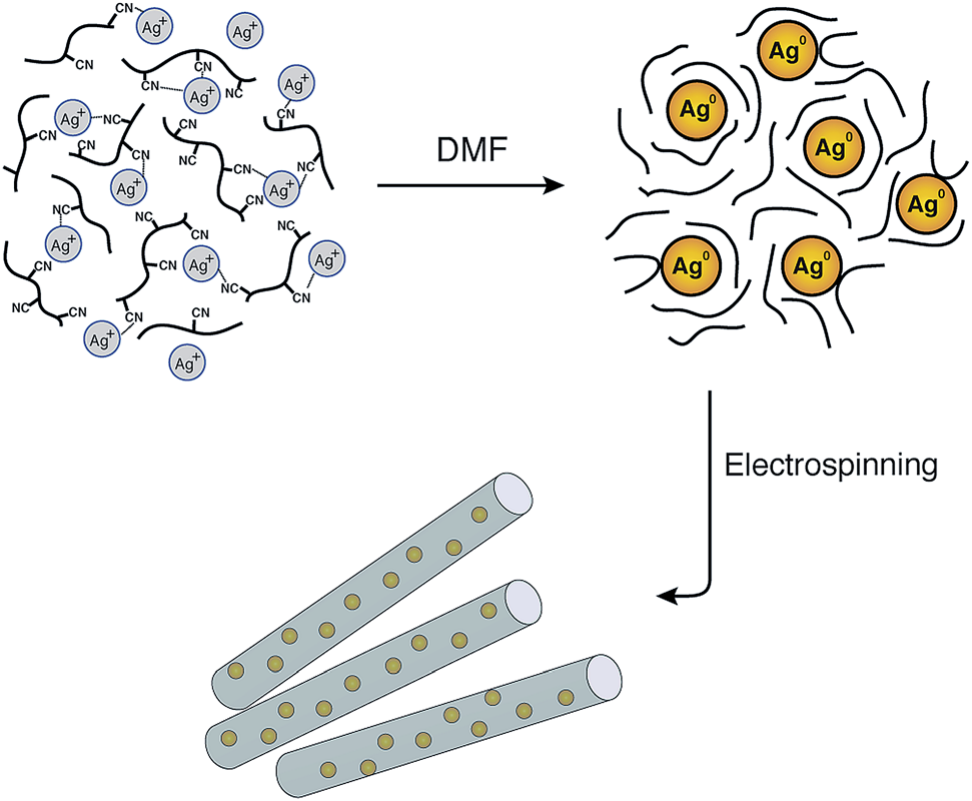
Schematic illustration of the formation of AgNPs/PAN composite nanofibres and the stability of the nanoparticles by means of polyacrylonitrile after reduction *via* DMF.

Although the reduction of Ag^+^ by DMF can be performed at room temperature, the reaction rate is very slow. Therefore, raising the temperature will increase the reduction rate. In addition, PAN may also behave as a stabiliser for the *in situ* formation of silver nanoparticles during the preparation of the spinning solution as shown in Fig. 1. The interactions between the silver nanoparticles and the nitrogen functional groups or the carbon-nitrogen triple bonds may prevent the agglomeration of Ag nanoparticles and promote the distribution of particles in the solution.

Several publications describe the use of Ag/PAN nanocomposite fibres for catalytic and antibacterial applications.^32–36^ In contrast, very few results have been reported on the use of CNFs as conductive materials. Park *et al.* studied the electrochemical behaviour of PAN/Ag-based carbon nanofibres using cyclic voltammetry (CV).^37^ In a similar manner Aussawasathien *et al.* prepared electrospun non-woven Ag-doped PAN-based CNFs and measured the conductive properties as a function of volume resistivity using the two-point probe method.^38^ Bei *et al.* also studied the fabrication of Ag/PAN composite nanofibres at low temperature followed by heat treatment at different temperatures.^39^ Hsieh *et al.* recently successfully produced electrospun silver ion-containing nanofibres from a solution of PAN/dimethylacetamide/tetraaniline (TeAN). Ag^+^ was reduced to atomic silver by TeAN after thermal treatment at 500 °C resulting in a highly transparent, conducting silver nanofibre (AgNFs) web.^40^

**Fig. 2:**
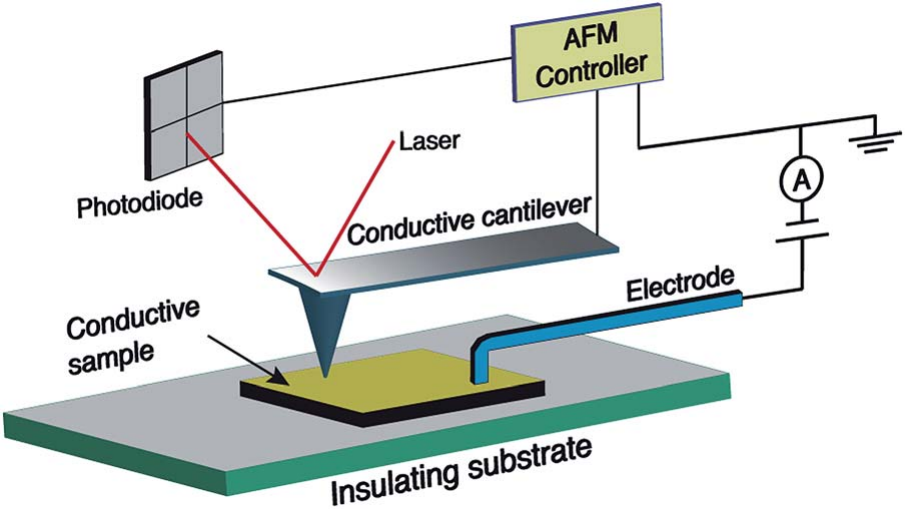
Geometry of the current sensing atomic force microscopy (CS-AFM) technique for measuring the in-plane current.

In spite of the wealth of scientific papers on the production and functionalisation of CNFs and their electrical properties, there is a certain lack of understanding of the relationship between structure and electrical properties. Thus, current-sensing atomic force microscopy (CS-AFM) as a new technique was proven to be effective for the characterisation of electrical properties at the nanoscale.^41^ Generally, AFM senses the surface features by employing a microfabricated cantilever with a sharp tip. In the case of CS-AFM, a conductive cantilever is used and an electrode is positioned at a fixed point of the sample as shown in Fig. 2. The conductive probe is scanned over a surface and the current resulting from a voltage applied between the probe and the electrode is recorded. The possibility of decoupling the force feedback, which controls the tip height, from the current feedback allows the analysis of both surface topography and electrical conductivity simultaneously. Hence, relationhips between the structural features and the electrical properties of the sample can be derived, which is not possible using other techniques.^42–45^

In this study, for the first time, the effect of the incorporation of AgNPs on the electrical conductivity of PAN-based CNFs is studied by the described CS-AFM technique. Accordingly, PAN nanofibres with different concentrations of AgNPs were electrospun, stabilised and carbonised to form AgNP-doped CNFs. The reduction of Ag^+^ into metallic AgNPs was performed directly in the PAN solution (DMF) prior to electrospinning. The influence of the AgNP concentration as well as the electrospinning parameters on the formation of nanofibres and their electrical properties were evaluated.

## Experimental section

### Materials

Polyacrylonitrile was donated by Dralon GmbH, Germany. Silver nitrate (AgNO_3_, 99.99%) and *N*,*N*-dimethylformamide (DMF, 99.8%) were purchased from Roth (Germany). All of these reagents were used as received without further purification.

### Preparation of PAN and AgNPs/PAN solutions

PAN spinning solutions were prepared in four different concentrations (6, 9, 12 and 15 wt%) by dissolving the required amount of PAN in 10 mL DMF. The mixture was stirred for 1 h at 60 °C and then 8 h at room temperature until light yellow homogeneous solutions were obtained. Spinning solutions containing AgNPs were prepared by adding silver nitrate to the PAN solution in concentrations of 20, 30, and 40 mmol per 10 mL. The PAN concentrations were 9 and 12 wt% in DMF in these cases.

### Electrospinning

The spinning solutions were loaded in a 5 mL syringe with a 24-gauge (26 G) stainless steel needle. The solutions were then fed through the needle using a syringe pump (KDS 100 model, K.D. Scientific Inc., Holliston, MA, USA) at a rate of 0.4 mL h^−1^. The tip of the needle was connected to a high voltage power supply (Heinzinger PNC 30000-5 ump, Heinzinger, Germany). Aluminium foil (20 cm × 20 cm) was used as a collector. The PAN solutions were electrospun at different applied positive voltages (15, 20 and 30 kV) with a 20 cm distance between the tip of the needle and the collector. The distance between the collector and the tip of the needle was also varied (10, 20 and 30 cm) at an applied voltage of 20 kV.

### Fabrication of CNFs and CNFs containing AgNPs

The PAN nanofibre mat was placed in a tube furnace with a gas supply system (Nabertherm R50/250/12, Lilienthal, Germany). The furnace temperature was increased to 270 °C with a heating rate of 15 K min^−1^ and the samples were stabilised in air for 90 min at this temperature. Afterwards, the temperature was increased to 650 °C with a heating rate of 13 K min^−1^ and the samples were held for an additional 60 min under a N_2_ flow to obtain carbon nanofibres.

### Characterisation and measurements

UV-visible absorbance spectra for the PAN and AgNPs/PAN precursor solutions as well as for the AgNPs in DMF were obtained using a spectrometer (Lambda 950 S, Perkin Elmer) in the region of 200 to 600 nm.

Dynamic light scattering was performed to determine the size distribution of the silver nanoparticles using a Zetasizer 1000 Malvern apparatus. Measurements were made at an angle of 90° and a temperature of 25 °C.

The surface tension of the precursor solutions was measured using a ring tensiometer (Dynometer plus, DY 21.1, BYK-Gardner, Germany).

The share viscosity of the precursor solutions was measured using a rheometer with a 50 mm cone-plate geometry (Rheometer Physica MCR-301, Anton Paar, Graz, Austria). The experiments were performed at a shear rate ranging from 0.01 to 50 s^−1^ at a temperature of 20°. A solvent trap was used to avoid evaporation during the measurements.

The conductivity of the precursor solutions was recorded using a conductometer (inoLab Cond Level 1, WTW GmbH & Co. KG, Germany).

The morphology of the electrospun PAN and AgNPs/PAN nanofibre web was observed using a scanning electron microscope (SEM S-3400 N II, Hitachi High-Technologies Europe GmbH) at different magnifications after surface sputtering with gold in a vacuum for 4 min using a Quorum Emitech K500X sputter coater (Ashford, Kent, UK). The diameter of the nanofibres (∼50 line-cuts for each image) was determined using ImageJ analysis software (public software produced by Wayne Rasband, the National Institute of Health). The average fibre diameter was plotted as a histogram, which was fitted to a Gaussian function using the Igor Pro 6 software tool (ESI, Fig. S1†). The elemental composition of the nanofibres was analysed using an energy-dispersive spectrometer (EDX) attached to the SEM.

The electrospun composite AgNPs/PAN nanofibres were analysed using TEM (FEI Tecnai F20) at 200 keV. The size distribution histogram of the silver nanoparticles was obtained by measuring the particle diameter in the TEM image using ImageJ analysis software.

CS-AFM measurements were performed in contact imaging mode using an Agilent Technologies 5500 AFM with beam deflection. NanoWorld contact-mode cantilevers with conductive PtIr5 coated silicon tips (force constant 2.8 N m^−1^, length 240 μm, mean width 35 μm and a thickness of 3 μm, and tip height 10–15 μm) were used. The CS-AFM nose assembly was used with a maximum measurable current of 1 nA. Therefore, the applied bias was adjusted during a single scan frame so that the maximum current was not exceeded. Bias voltage was directly applied to the sample using a conductive electrode (see Fig. 2). The sampling resolution of all the images was set at 1024 data points with a scanning speed of 1 line per second. All of the captured images were processed by the open-source Gwyddion software.

CS-AFM measurements were carried out for the CNF samples obtained from the electrospun PAN and composite AgNPs/PAN nanofibres at different PAN and Ag^+^ ion concentrations, while the applied voltage and the distance between the tip of the needle and the collector were fixed at 20 kV and 20 cm, respectively.

## Results and discussion

### Optimisation of the electrospinning parameters for PAN nanofibres

The diameter, and hence the properties of the electrospun nanofibres, largely depends on the viscosity of the PAN precursor as well as the applied voltage during the electrospinning process.^46^ Therefore, the first task of this study consisted of optimising the concentration of the PAN precursor and the electrospinning voltage before incorporating the silver nanoparticles. Thus, the morphology of the nanofibres with different PAN concentrations (6, 9, 12 and 15 wt%) and under various applied voltages (15, 20 and 30 kV) was investigated.

**Fig. 3:**
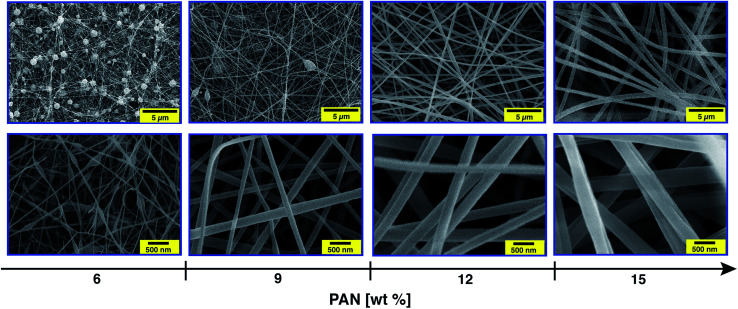
SEM images of the electrospun PAN nanofibres produced from solutions with different PAN concentrations (6, 9, 12 and 15 wt%). The applied voltage and the distance between the tip of the needle and the collector were fixed at 20 kV and 20 cm, respectively. The scale bar is 5 μm (top) and 500 nm (below).

Fig. 3 clearly shows that the utilisation of a 6 wt% PAN concentration mainly resulted in the formation of droplets, which was observed for all of the studied voltages in this work. At a concentration of 9 wt% only a few beads were formed, while a uniform nanofibre structure with extensive chain entanglement was obtained using a 12 wt% PAN solution.

The concentration effect can be explained in terms of viscosity and surface tension of the polymer solution, which significantly affect the uniaxial stretching of the charged jet. A rapid non-linear increase in the viscosities of the PAN precursor solutions was observed with increasing PAN content, while no dramatic change in the surface tension was noted (ESI, Fig. S2†). For solutions with a low PAN concentration, the entangled polymer chains cannot overcome the surface tension after applying the electrical field. This leads to fragmentation of the polymer chains before reaching the collector.^47^ Thus, the flow of the fluid jet cannot be elongated to form oriented fibres. Instead, polymeric particles or a mixture of beads and fibres or beaded nanofibres will be formed. As the viscosity of the PAN solution is above a critical point, which can be tuned by adjusting the polymer concentration, smooth continuous nanofibres can be obtained. Increasing the concentration to 15 wt%, fibres with a uniform structure were again obtained. In addition, increasing the solution concentration from 6 to 15 wt% yielded in gradual increasing of the fibre diameter (ESI, Fig. S3†). For instance, the average diameter of the electrospun fibres with a concentration of 9 wt% was about 110 nm, while that of 12 wt% was ∼240 nm. Moreover, the full width at half maximum (FWHM) of Gaussian shaped peaks also increased as the concentration of the PAN solution increased, indicating broadening in the diameter distribution. However, the average diameter of the nanofibres using the 6 wt% solution was calculated excluding the beads.

The applied voltage is an important process parameter that affects the morphology and diameter of the nanofibres. The average diameter of the electrospun fibres for three different PAN concentrations increased slightly (by ∼30 nm) as the voltage increased from 15 to 20 and 30 kV (ESI, Fig. S4†). A differing behaviour was observed only in the case of the fibres obtained at high concentration, where a notable increase in diameter occurred when the voltage was increased from 20 to 30 kV. It has to be noted that controversial results have been reported in this respect in the literature. Some groups found totally opposite behaviour and explained this with electrostatic repulsive forces on the jet which increased with the applied voltage.^46^

Additionally, other working parameters can also affect the diameter of the nanofibres, namely the type of electrospun polymers and their solution conductivity, the flow rate, the type of collector and the distance between the collector and the tip of the syringe. The effect of the last factor was also investigated in this study, and we found that an increase of the fibre diameter from ∼120 to 150 nm was associated with an increase of the spinning distance from 10 to 30 cm (ESI, Fig. S5†). This would be related to a reduced stretching as the electric field decreases with increasing distance between the collector and the tip of the syringe. The FWHM was increased, indicating the formation of fibres with a wide range of diameters. This may be because the fibres are solidified at different times owing to the long distance.

It is worth noting that many research groups have also studied the effect of solution conductivity on the formation of the Taylor cone as well as controlling the diameter of the nanofibers.^48^ Increasing the conductivity of the electrospun solution to a critical value causes an increase in the surface charge of the droplet, resulting in the formation of the Taylor cone and a drop in the fiber diameter.^49^ Therefore, the conductivity of the electrospun solutions as a function of PAN concentration was also investigated in this study (ESI, Fig. S6†). Although an increase in the conductivity with increasing polymer concentration was observed, this increase could not overcome the effect of the viscosity term, which yields an increase in the fiber diameter as we discussed above.

### Electrospinning of the AgNPs/PAN composite nanofibres

**Fig. 4:**
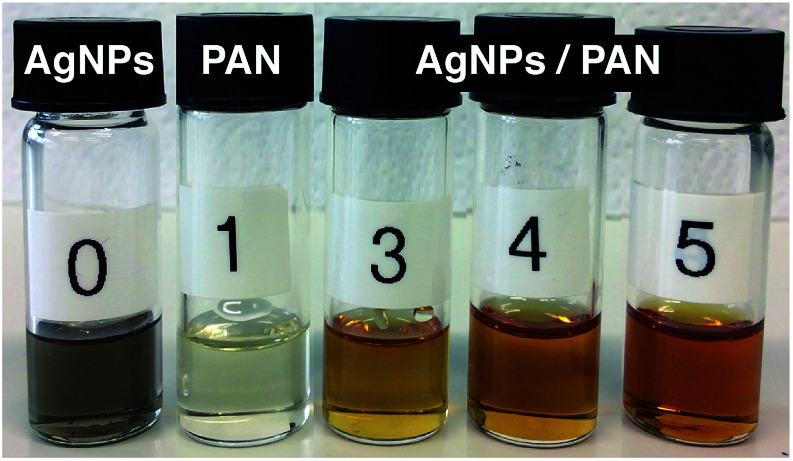
Photographs representing: (0) the formation of metallic Ag nanoparticles in DMF solution; (1) the polyacrylonitrile (PAN) solution in DMF (12 wt%); and ((3)–(5)) formation of AgNPs within the PAN solutions (12 wt%) at different Ag^+^ ion concentrations: (3) 2.6, (4) 3.9 and (5) 5.2 wt% with respect to PAN. All solutions were heated for 1 h at 60 °C and then stirred overnight at room temperature.

The formation of AgNPs within the PAN solutions was visually proved by a yellow-brown colour occurring after heating the solution for 1 h at 60 °C (Fig. 4). The colour gradually became darker and intensified with an increasing concentration of Ag^+^ and the related increase in the AgNP amount.

**Fig. 5:**
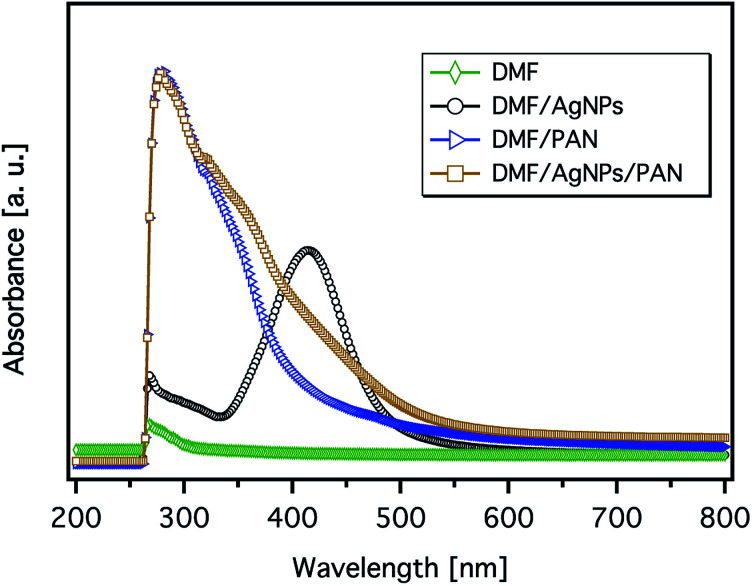
The UV-Vis spectra of the 9 wt% PAN solution, metallic Ag nanoparticle formation in DMF (40 mmol AgNO_3_), and their corresponding mixture (the concentration of AgNO_3_ is 5.2 wt% with respect to PAN).

The formation of AgNPs was also confirmed by measuring the UV-Vis absorbance of the solutions, and the representative spectra are shown in Fig. 5. The pure AgNP dispersion in DMF clearly shows the expected absorption band with a peak maximum at 414 nm, which relates to the characteristic surface plasmon resonance (SPR) of metallic Ag nanoparticles with a diameter below ∼10 nm at a wavelength around 400 nm.^35^ However, the broadness of the spectrum and the shift of its peak towards a higher wavelength indicate the formation of particles with bigger diameters. The spectrum of the PAN solution shows an intense band with a sharp maximum at ∼280 nm and a weak shoulder band at a wavelength of ∼318 nm. It can be also seen from Fig. 5 that the spectrum of the 9 wt% PAN solution containing 5.2 wt% AgNO_3_, with respect to PAN, exhibits an additional broad shoulder above 350 nm, which can be attributed to the AgNPs. However, there is a remarkable blue shift of the energy transition induced by the AgNPs. This observation indicates that the size of the nanoparticles in the presence of PAN is smaller than that without polymer chains. The overall effect can be explained due to the stabilisation of the formed Ag nanoparticles with PAN. On the other hand, Lee *et al.* reported that the intensity of the SPR of the Ag particles in the presence of PAN increased with time without any shift in the value of the maximum absorbance even after 7 days.^31^ In other words, the average size of the formed Ag nanoparticles was not increased, which may be attributed to the stabilisation of the particles by polymer chains.

In this context, the size distribution of the formed nanoparticles was evaluated using photon correlation spectroscopy (PCS, alternatively known as the dynamic light scattering technique, DLS). Silver nanoparticles prepared by the oxidation of DMF reveal an average diameter of ∼40 nm with a narrow size distribution. The average size of the particles in the presence of PAN increases up to 180 nm with a large FWHM compared to that of pure AgNPs (ESI, Fig. S7†). The increase of the average nanoparticle size with significant peak broadening is attributed to the strong interaction of the polymer molecule with the surface of the silver nanoparticles. A typical interaction pattern involves bonding of the polymer end-chain with the surface of AgNPs. The modes of interaction are mainly correlated with the charge nature as well as the molecular weight of the polymer. However, linear polymer chains with high molecular weights usually undergo the so-called tail mode of interaction.

**Fig. 6:**
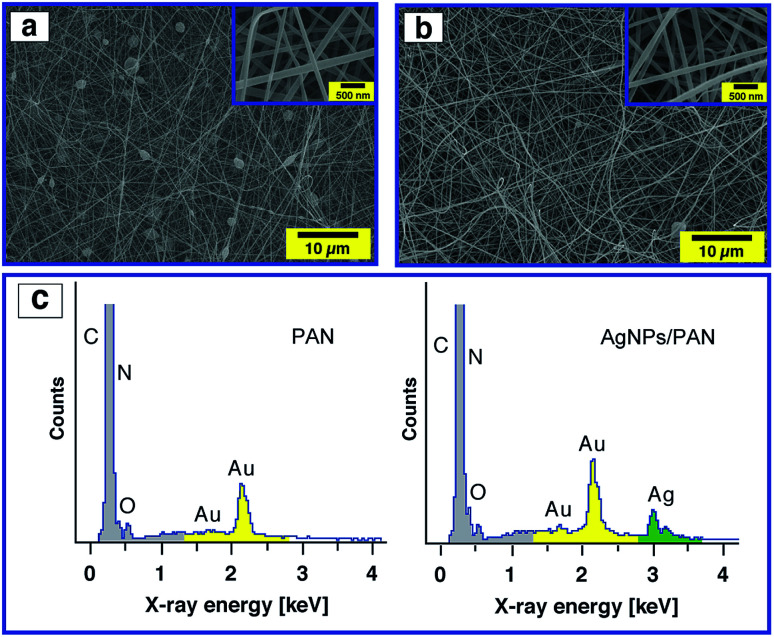
SEM images of the electrospun PAN nanofibres (9 wt%) without (a) and with (b) Ag nanoparticles (5.2 wt% with respect to PAN) at two different magnifications (inset), and their corresponding SEM-EDX spectra (c). The applied voltage and the distance between the tip of the needle and the collector for all data were fixed at 20 kV and 20 cm, respectively.

The surface morphology of the AgNPs/PAN composite nanofibre webs was characterised by SEM. In contrast to pure PAN-based nanofibres, the composite nanofibres were bead-free and continuous at the same PAN concentration of 9 wt% (Fig. 6a and b). The average diameter of the composite fibres was found to be greater than that of the pure fibres without AgNPs with a broad size distribution (ESI, Fig. S8†). It was also observed that the average diameter of the composite fibres increased with an increasing concentration of AgNO_3_ (ESI, Fig. S9†). Both the viscosity and the conductivity of the electrospun solutions were increased as a function of AgNO_3_ concentration, while the surface tension remained the same. Therefore, the increase in fiber size can indeed be explained by an increase in the viscosity of the precursor solution resulting from polymer–particle interactions (ESI, Fig. S10 and S11†). The presence of Ag in the composite fibres was principally confirmed by EDX as shown in Fig. 6c.

**Fig. 7:**
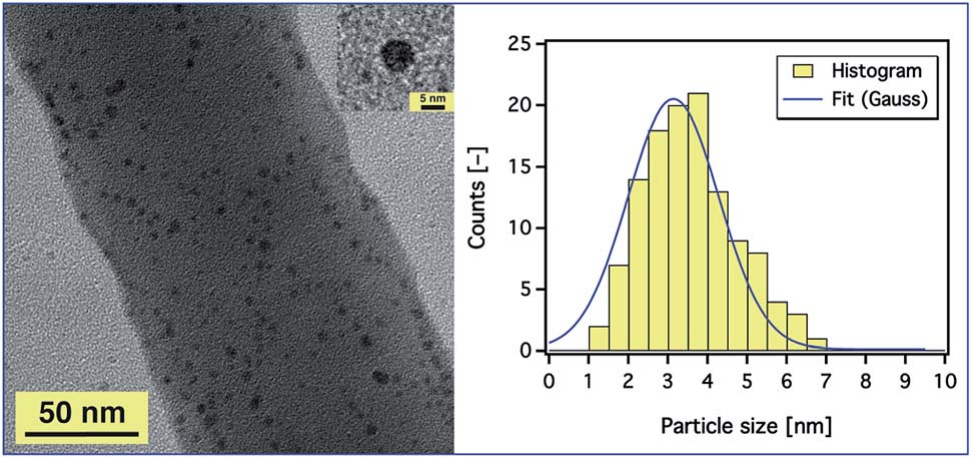
A TEM image of PAN nanofibres electrospun from a 9 wt% PAN solution containing 5.2 wt% AgNO_3_ with respect to the PAN concentration (left). The inset is a silver nanoparticle with a diameter of ∼6 nm. (Right) A histogram of the size distribution of the Ag nanoparticles with Gaussian fit. The applied voltage and the distance between the tip of the needle and the collector were fixed at 20 kV and 20 cm, respectively.

In order to prove the distribution of AgNPs, a magnified SEM image was also obtained (ESI, Fig. S12†), but we could not observe any aggregation as SEM studies only the surface structure of the fibres. Therefore, TEM studies were conducted to get an insight into the dispersion of AgNPs inside the composite nanofibres. Fig. 7 (left) shows a TEM image of the PAN fibres electrospun from a 9 wt% PAN solution containing 5.2 wt% Ag^+^ with respect to PAN. It is observed that the AgNPs were sphere shaped (see Fig. 7, inset) and well distributed on and within the fibres. The average diameters of the particles as measured from the TEM micrographs were in the range of 1 to 7 nm as shown in Fig. 7 (right), which enhances and confirms the results obtained from the UV-Vis absorption spectra and our assumption that the AgNPs are stabilised by PAN polymer chains. Also, the diameter of the composite fibres was found to be ∼130 nm, which is consistent with the results obtained from SEM.

### AgNPs/PAN-based CNFs and their conductive properties

PAN- and AgNPs/PAN-based carbon nanofibres were prepared by a two-step heat treatment of the electrospun fibres. The as-spun nanofibre samples were peeled off the aluminium foil and placed on a silicon wafer before heat treatment in a horizontal tube furnace. The stabilisation process was conducted at 270 °C for 90 min in air. Subsequently, the samples were carbonised under a N_2_ flow at 650 °C for 60 min. AFM was used to observe the topography and friction of the CNFs after both stabilisation and carbonisation.

**Fig. 8:**
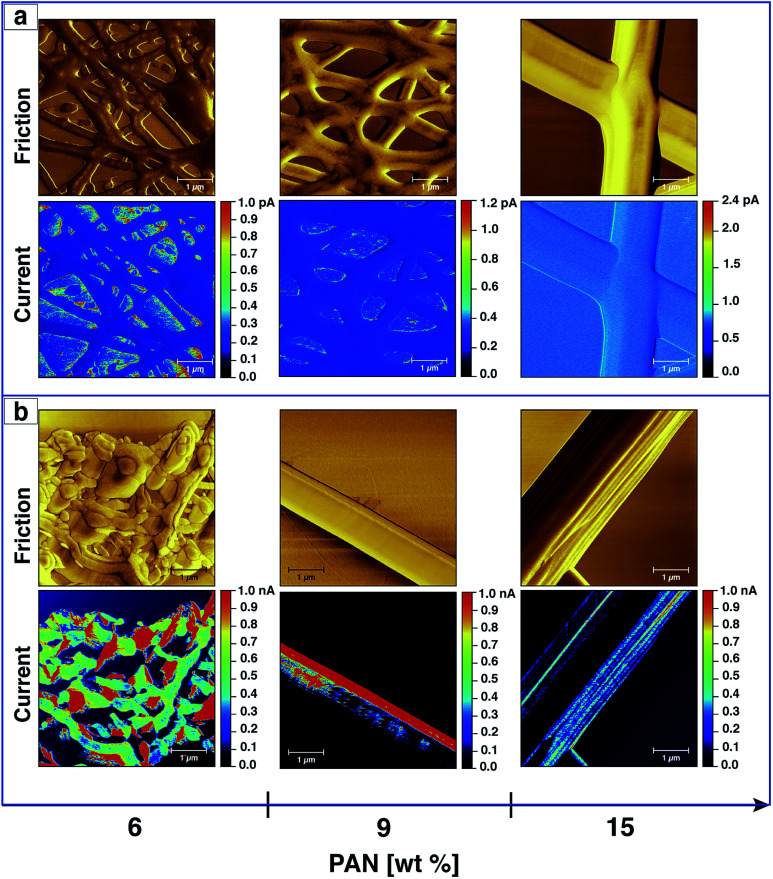
CS-AFM analysis of CNFs processed from PAN nanofibres electrospun with different concentrations. Images show the friction and current after both stabilisation (a) and carbonisation (b) processes. The applied bias voltage was +0.15 V. The scan area was 5 × 5 μm^2^ with a scale bar of 1 μm.

Fig. 8 displays the friction of the fibres without Ag nanoparticles after the stabilisation and carbonisation processes. It can be clearly seen that the diameter of the fibres increase as they melt together after the heating treatment, which is related to the formation of a web-type structure upon electrospinning. A mixture of beads and fibres were also observed if PAN solutions of a low concentration were utilised. However, no major change in morphology was detected after the carbonisation process in comparison with that after stabilisation, except for the fibres electrospun from a 6 wt% solution, which undergo further melting resulting in structural damage as shown in Fig. 8. However, the merging of the fibers upon heat treatment unfortunately prevent us from studying the relationship between the morphologies of AgNPs/PAN fibres and their conductive properties (ESI, Fig. S13†).

**Fig. 9:**
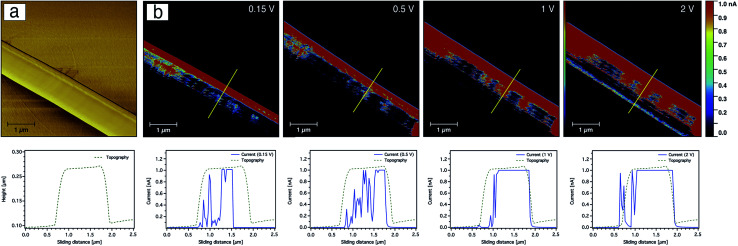
CS-AFM images of friction (a) and current (b) from the 9 wt% PAN-based-CNFs without AgNPs at various bias voltages. The yellow line marks the area where the line profiles for both topography and current are shown below the current images. The scan area was 5 × 5 μm^2^ with a scale bar of 1 μm.

In addition to probing the topography, CS-AFM has the advantage of measuring and mapping the local electrical properties of individual carbon nanofibres at the nanometer-scale of current measurement. As expected, CS-AFM measurements of stabilised nanofibres yielded no current as shown from the 2D current maps in Fig. 8, even at high bias voltage (data not shown). However, after carbonisation a current can be clearly observed indicating the formation of PAN-based CNFs. The 2D current maps of the fibres after carbonisation do not show current saturation (1 nA) over the whole fibre area at an applied bias voltage of 0.15 V. This can be attributed either to the joining/blending of the nanofibres during the heat treatment resulting in fibres which are not equally carbonised, or to the incomplete carbonisation process. Fig. 9 shows the current evaluation of the CNFs without AgNPs as the bias voltage was ramped from 0.15–2 V. One can easily observe that the current saturation area of the scanned fibre increased as the bias voltage increased. At a 2 V bias voltage, almost all of the fiber areas exhibit a current higher than the upper detection limit as we see from the line profiles of both topography and current below the AFM images in Fig. 9.

**Fig. 10:**
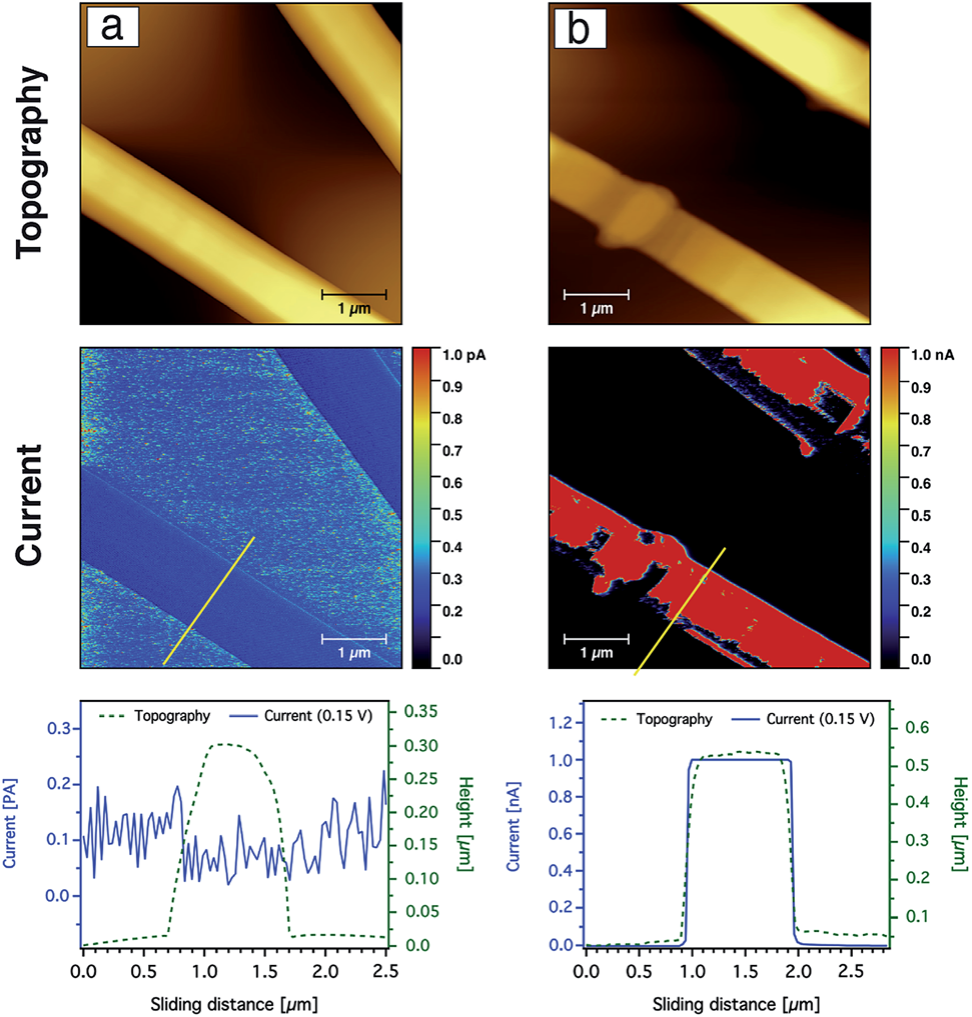
CS-AFM analysis of CNFs processed from PAN nanofibres (9 wt%) containing silver nanoparticles (40 mmol) after both stabilisation (a) and carbonisation (b). Images show topography and current after both stabilisation and carbonisation processes. A yellow line marks the area where the line profiles for both topography and current are shown below. The applied bias voltage was +0.15 V. The scan area was 5 × 5 μm^2^ with a scale bar of 1 μm.

The current sensing images of the carbon nanofibres containing Ag nanoparticles are shown in Fig. 10. In contrast to the CNFs without AgNPs, the current saturation over the total scanned area of the fibre was already observed at a bias voltage of +0.15 V as shown from the topography and current line profiles. This indicates that the electrical conductivity of the CNFs was significantly enhanced by the incorporation of silver nanoparticles. In addition to the electrical properties of the AgNPs, such behaviour or enhancement could be also related to the fact that the diffusion of heat during the stabilisation as well as the carbonisation processes throughout the CNFs are more uniform due to the thermal conductivity of the silver nanoparticles. Moreover, the nanoparticles may create channels for heat diffusion between the inner part and the surface of the CNFs during the heat treatment.

**Fig. 11:**
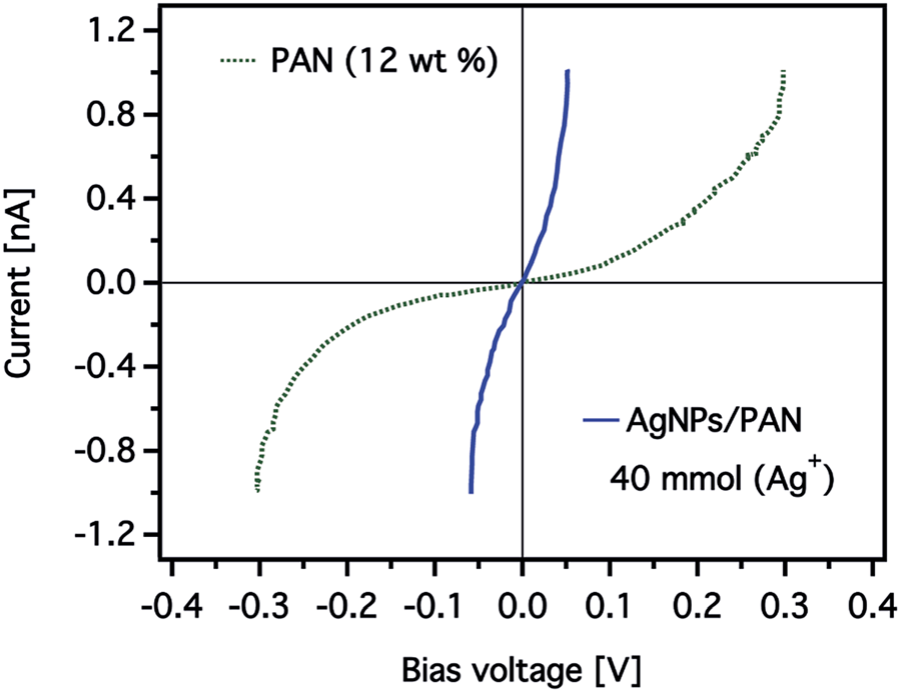
Representative current–voltage (*I*–*V*) curves obtained from CNFs prepared from electrospun PAN nanofibres (12 wt%) without (the green dashed line) and with (the blue solid line) AgNPs (40 mmol). The total current measured was over a 1 × 1 μm^2^ scan area of the CNFs.

The effect of the silver nanoparticles on the conductive properties can be also displayed more clearly by comparing current–voltage (*I*–*V*) curves obtained for the CNFs as shown in Fig. 11. The *I*–*V* curve was taken while scanning the bias voltage from −0.3 to +0.3 V. The detected current of the CNFs without AgNPs was nearly zero below a bias voltage of 50 mV, while a significant current was recorded above a threshold voltage of about 200 mV. In contrast, the *I*–*V* curve obtained from the composite nanofibres showed a strong increase from near zero voltage and current saturation was detected at a bias voltage of 50 mV.

**Fig. 12:**
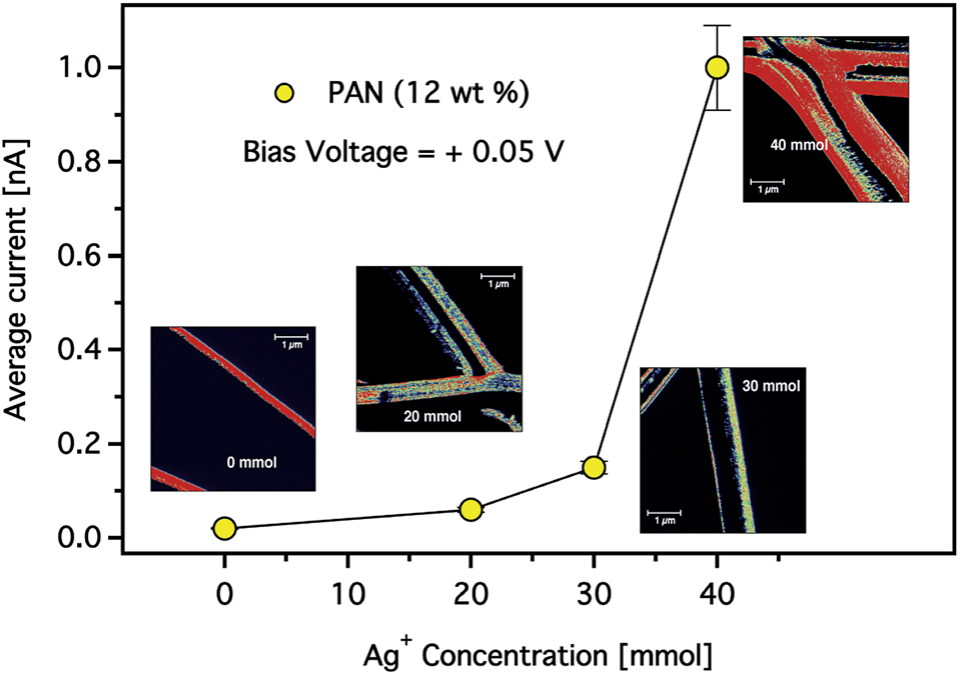
The total average current observed for the composite CNFs as a function of Ag^+^ concentration. (Inset) CS-AFM images of the electrospun CNFs with 12 wt% concentrations of PAN solution. The applied bias voltage was +0.05 V. The scan area was 5 × 5 μm^2^ with a scale bar of 1 μm.

The effect of the silver nanoparticle concentration on the electrical properties of the CNFs was also studied. Fig. 12 shows the average current at a constant bias voltage of +0.05 V in dependence on the Ag^+^ concentration in composite CNFs prepared using a solution of 12 wt% PAN. It is clearly shown that the total average current increased as the concentration of the Ag^+^ increased. For instance, at a constant voltage while the Ag^+^ concentration increased from 30 to 40 mmol, the current increased by a factor of five. It is worth noting that an increase in conductivity is observed despite the increase in the average diameter of the composite fibres. As we stated above, this observation may indicate that the electrical resistance of the carbon nanofibres decreases by introducing more AgNPs resulting in higher conductivity. Nevertheless, the mechanism of this conductivity enhancement should be carefully taken considering the various parameters associated with the formation of composite CNFs.

## Conclusions

Composite carbon nanofibres were prepared from a silver/polyacrylonitrile precursor using an electrospinning technique and subsequent heat treatments. The diameter distribution of the electrospun AgNPs/PAN nanofibres was influenced by various parameters such as the concentration of the PAN solution, the silver ion concentration and the applied voltage during the electrospinning process. In accordance with the PAN content and the spinning voltage, a uniform nanofibre morphology was obtained using a PAN solution of 12 wt% at 20 kV. The average diameter of the Ag/PAN composite nanofibres is greater than the as-spun PAN nanofibres. In addition, the AgNP concentration affects the fibre characteristics (fibre diameter and electrical properties). UV absorption spectroscopy, SEM-EDX and TEM demonstrated the formation of AgNPs with an average diameter of less than 4 nm. Moreover, the addition of AgNPs was found to be an effective means of improving the conductive performance of the CNFs. This enhancement in the electrical properties is dependent on the concentration of AgNPs. This composite material holds promise to be used as a new innovative highly conductive electrode for many potential applications including lithium-ion batteries or supercapacitors with a more optimised CNF structure, and detailed investigations are to follow. In particular, deeper insight into the underlying effects of the heat treatment during the stabilisation and carbonisation processes as well as the effect of the nanoparticle size on both the morphology and properties of the composite CNFs will be investigated in a subsequent study.

## Conflicts of interest

There are no conflicts to declare.

## Supplementary Material

RA-009-C8RA04594A-s001
