# Supplementary material for: Electrical conductivity of silver nanoparticle doped carbon nanofibres measured by CS-AFM
Source: RSC Adv. 2019 Feb 5;9(8):4553–62. doi: 10.1039/c8ra04594a (PMC9060595; doi:10.1039/c8ra04594a)
Supplement: RA-009-C8RA04594A-s001 [file RA-009-C8RA04594A-s001.pdf]

Electronic Supplementary Material (ESI) for RSC Advances.
This journal is © The Royal Society of Chemistry 2019

Electric conductivity of silver nanoparticle doped carbon nanofibres measured by CS-AFM

Wael Ali,[a,b] Valbone Shabani,[b] Matthias Linke,[b] Sezin Sayin,[b] Beate Gebert,[a] Sedakat Altinpinar,[b] Marcus Hildebrandt,[b] Jochen S. Gutmann*[a,b] and Thomas Mayer-Gall*[a,b]

[a] Deutsches Textilforschungszentrum Nord-West gGmbH, Adlerstr. 1, D-47798 Krefeld, Germany
[b] Department of Physical Chemistry and Center for Nanointegration Duisburg-Essen (CENIDE), University Duisburg-Essen, Universitätsstrasse 2, D-45141 Essen, Germany.

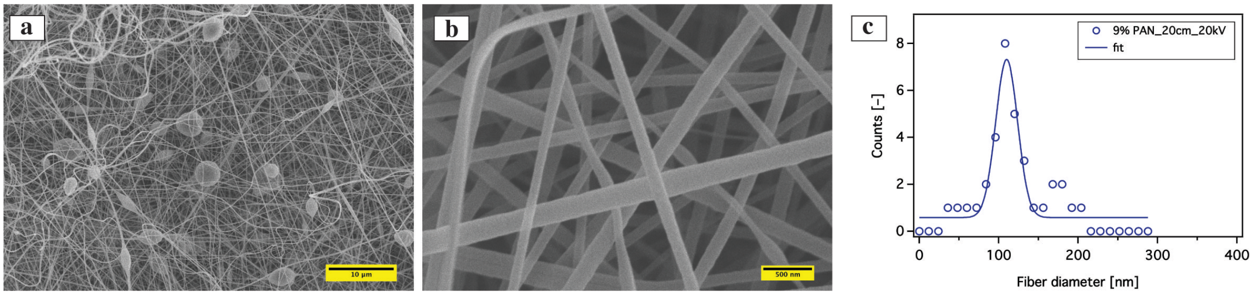

Fig. S 1 SEM images of electrospun PAN nanofibers (9 wt%) without Ag nanoparticles at two different magnifications (a, b). The applied voltage and the distance between the tip of the needle and the collector for all data were fixed at 20 kV and 20 cm, respectively. Graph (c) gives the diameter distributions of PAN nanofibers. The average fiber diameter was plotted as a histogram (∼50 line-cuts), which was fitted to Gaussian function using Igor Pro 6 software tool.

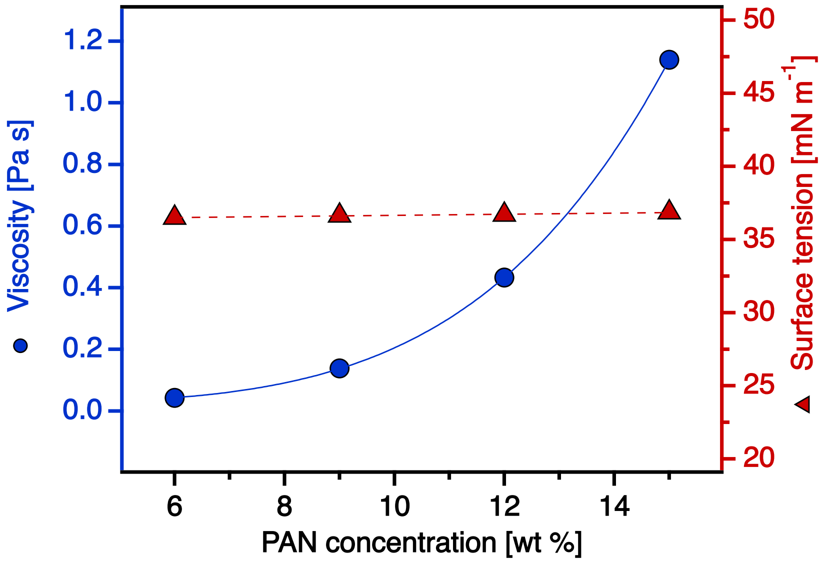

Fig. S 2 The variation of PAN solution viscosity and the surface tension as a function of the PAN concentration.

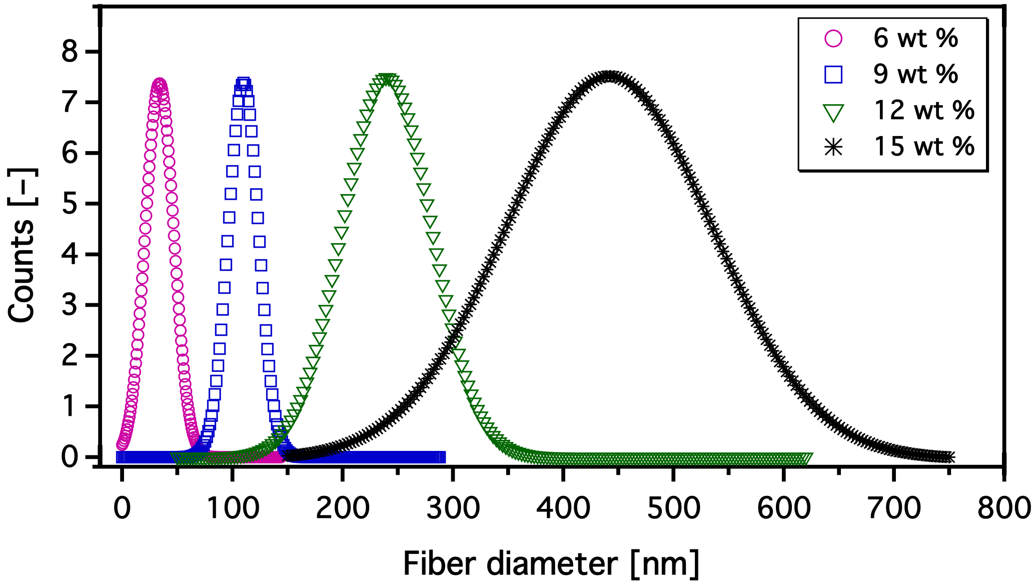

Fig. S 3 The average dimeters of nanofibers produced from solutions with different concentrations of PAN precursor solution. The applied voltage and the distance between the tip of the needle and the collector were fixed at 20 kV and 20 cm, respectively.

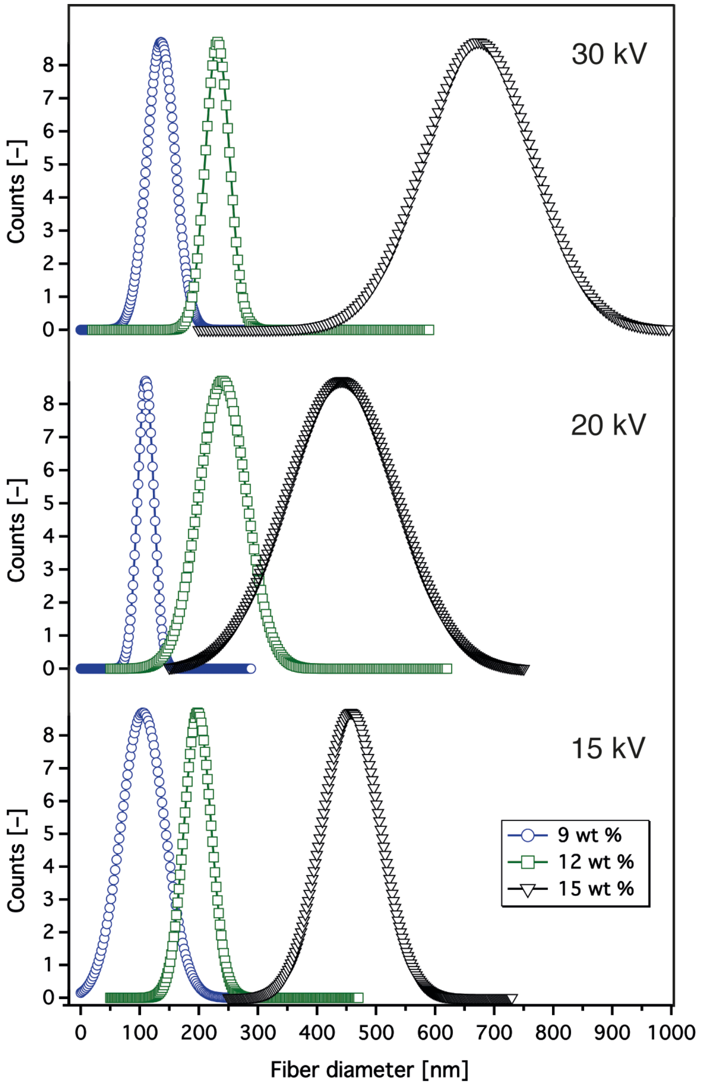

Fig. S 4 The average diameters of nanofibers produced from solutions with different PAN concentration and at different applied voltage during the electrospinning process. The distance between the tip of the needle and the collector was fixed at 20 cm.

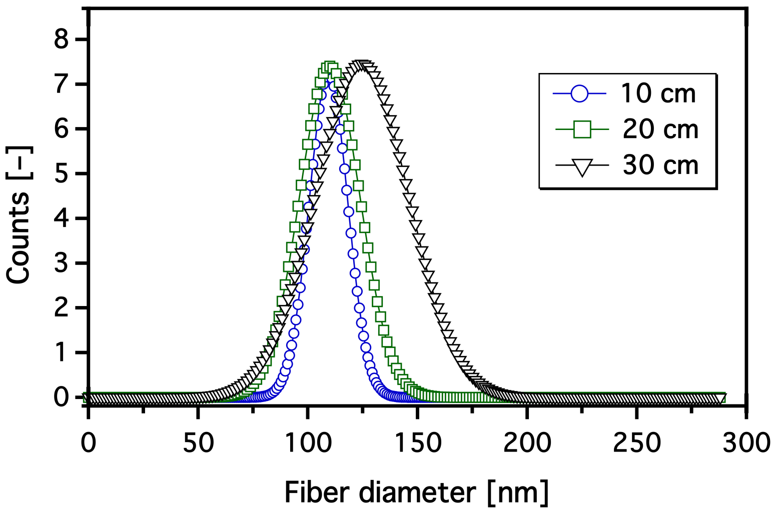

Fig. S 5 The average diameters of nanofibers spun at different distances between the collector and the tip of the syringe. The PAN concentration was 9 wt% and the applied voltage 20 kV.

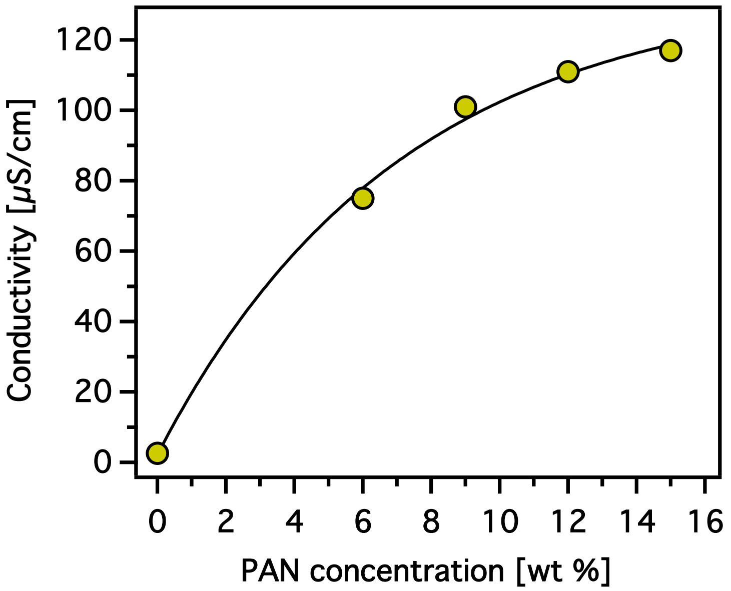

Fig. S 6 Electrical conductivity of PAN precursor solution as a function of PAN concentration.

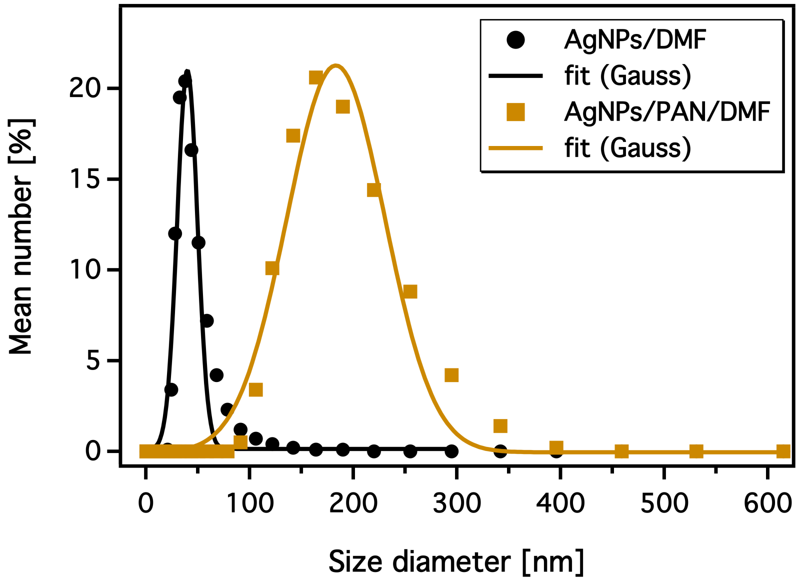

Fig. S 7 Size distribution of Ag nanoparticles synthesized using DMF as reduction agent without and with 9 wt% PAN. The concentration of the $AgNO_3$ is 40 mmol and 5.2 wt% with respect to PAN.

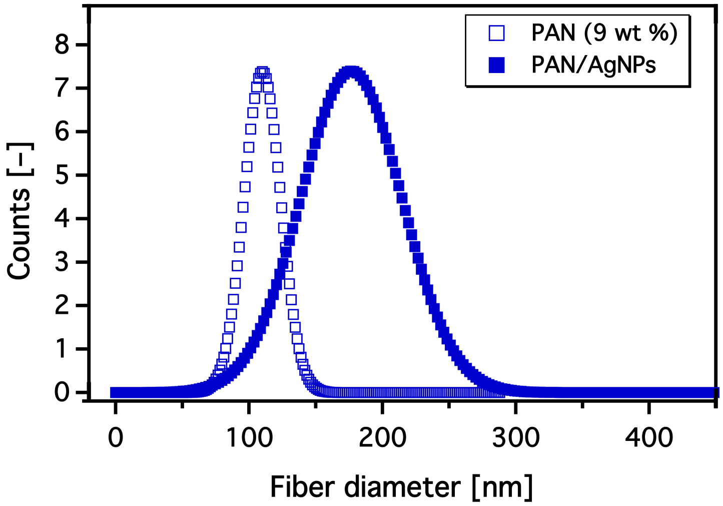

Fig. S 8 The diameter distributions of electrospun PAN nanofibers (9 wt%) without and with Ag nanoparticles (5.2 wt% with respect to PAN). The applied voltage and the distance between the tip of the needle and the collector for all data were fixed at 20 kV and 20 cm, respectively.

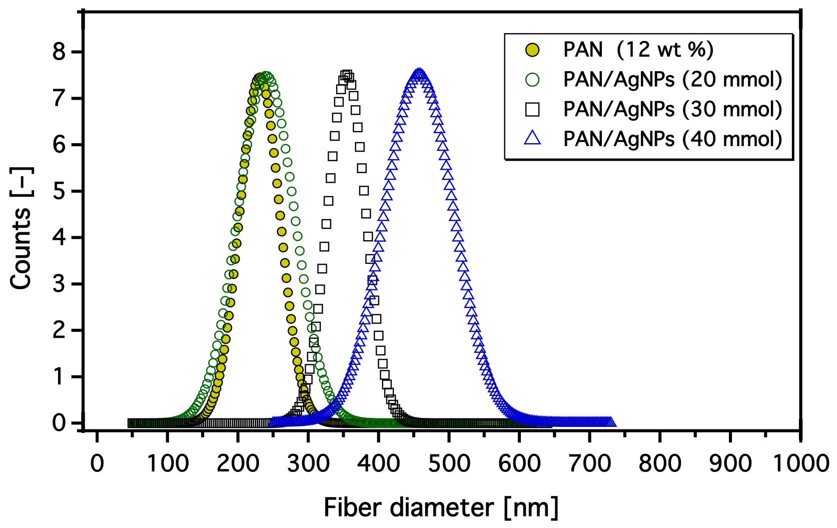

Fig. S 9 The effect of Ag^+ concentration on the fiber diameter of the PAN composite nanofibers (9 wt% PAN and 5.2 wt% Ag nanoparticles with respect to PAN). The applied voltage and the distance between the tip of the needle and the collector for all data were fixed at 20 kV and 20 cm, respectively.

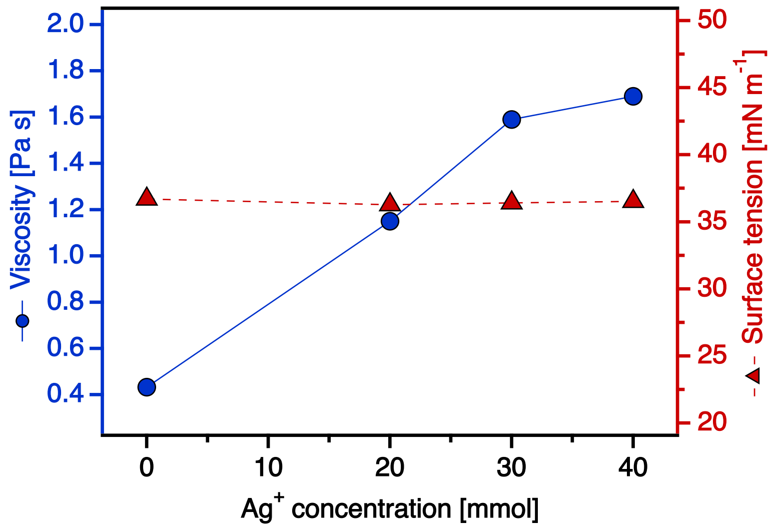

Fig. S 10 The variation of $AgNO_3$/PAN solution viscosity and the surface tension as a function of Ag^+ concentration. The PAN concentration is 12 wt%.

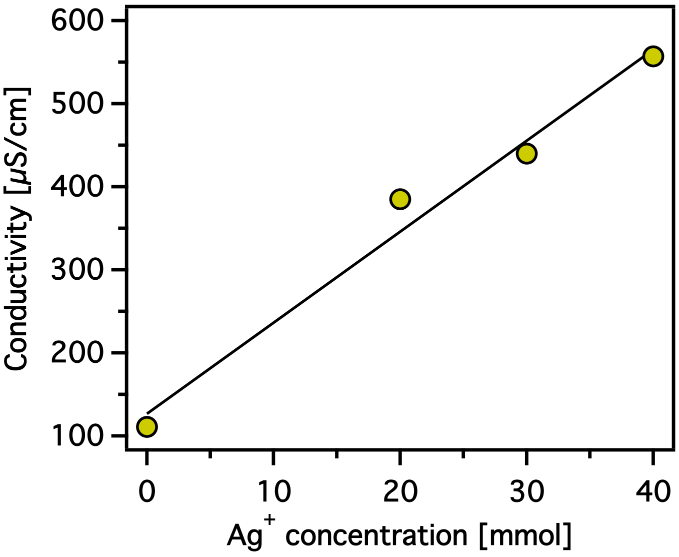

Fig. S 11 Electrical conductivity of $AgNO_3$/PAN precursor solution as a function of Ag^+ concentration. The PAN concentration is 12 wt%.

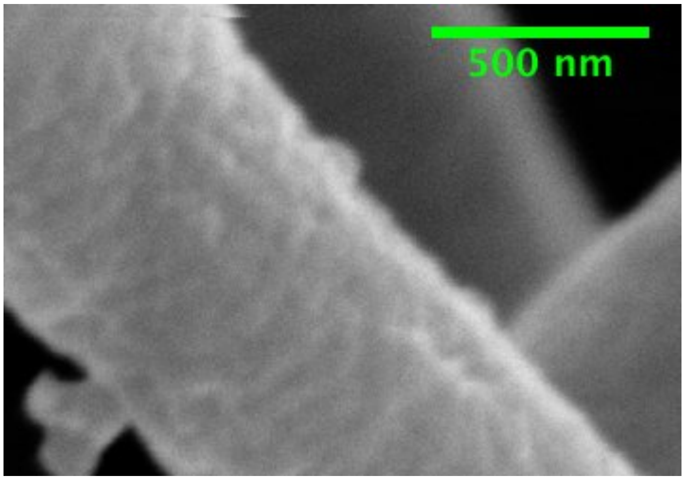

Fig. S 12 Magnified SEM image of electrospun $AgNO_3$/PAN composite nanofibers with 12 wt% PAN concentration and 40 mmol of $AgNO_3$. The applied voltage and the distance between the tip of the needle and the collector were 20 kV and 20 cm, respectively.

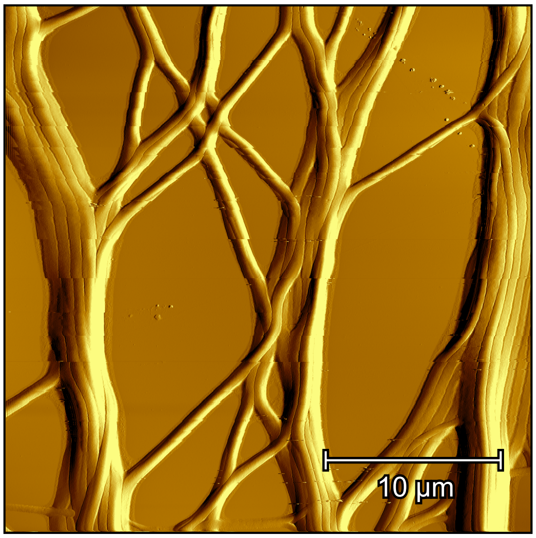

Fig. S 13 AFM friction image of electrospun $AgNO_3$/PAN composite nanofibers after carbonisation showing the merging of the fibres upon heat treatment.
